# A Survey on Knowledge, Attitude, and Current Practice in Diabetic Foot Care Among Indian Physiotherapists

**DOI:** 10.1155/tswj/3077222

**Published:** 2026-06-20

**Authors:** Niharika R. Kunder, Vijay Pratap Singh, K. Vijaya Kumar

**Affiliations:** ^1^ Department of Physiotherapy, Kasturba Medical College Mangalore, Manipal Academy of Higher Education, Manipal, India, manipal.edu

**Keywords:** attitude, diabetic foot, healthcare, India, knowledge, physical therapists, practice patterns

## Abstract

**Background:**

Diabetic foot ulcers are a major complication of diabetes and contribute significantly to morbidity and healthcare burden in India. Physiotherapists play a vital role in prevention and management; however, their knowledge, attitude, and clinical practices in the Indian context remain unexplored.

**Objective:**

The study is aimed at assessing the knowledge, attitudes, and practices (KAP) of physiotherapists practicing across India regarding diabetic foot care through a national online cross‐sectional survey.

**Method:**

A cross‐sectional national online survey was conducted from January 2024 to March 2025 among physiotherapists practicing across India. A total of 249 participants were included using a convenience sampling technique. Data were collected using a self‐developed, content‐validated questionnaire consisting of 20 questions across knowledge, attitude, and clinical practice domains. Descriptive statistical analysis was performed using Jamovi (2.6.23).

**Results:**

A total of 249 respondents from 28 Indian states were included. While 94.8% acknowledged their role in diabetic care, only 41% had received formal training in diabetic foot management. Although most participants identified clinical signs of complications, inconsistencies were observed in interpreting ABI and HbA1c values. Although 98.8% reported providing patient education practices, only 47.4% performed routine foot assessments. Greater clinical experience was associated with better application of knowledge in practice.

**Conclusion:**

Indian physiotherapists demonstrate positive attitudes toward diabetic foot care; however, gaps remain in formal training and consistent clinical practice persist. Strengthening structured education and developing standardized clinical guidelines are essential to improve practice.

## 1. Introduction

Diabetes is a long‐term metabolic condition characterized by persistently blood glucose levels, contributing significantly to global mortality and disability across all demographics [1, 2].

According to the International Diabetes Federation (IDF) 2024 report, India has a total adult population of approximately 947.4 million, among whom 10.5% are affected by diabetes, corresponding to an estimated 89.8 million adults living with diabetes.

This highlights India′s critical role in the global diabetes crisis and its significant contribution to diabetes‐related disability‐adjusted life years (DALYs) [3].

The disease manifests in four primary types. Type 1 diabetes results from autoimmune destruction of insulin‐producing cells; this contrasts with Type 2, where insulin secretion loss and resistance are key features [4]. Other specific types are associated with genetic defects, pancreatic disorders, or drug‐induced causes, while gestational diabetes, diagnosed during pregnancy, completes the quartet. Amid this diversity, tailored management approaches are essential. The American Diabetes Association stipulates that a fasting plasma glucose level of 126 mg/dL or higher (7.0 mmol/L) confirms diagnosis [5].

The escalating annual rate of diabetes diagnosis is projected to substantially increase the prevalence of diabetic foot, a complication characterized by deep tissue damage, infection, or ulceration in the lower limbs, often associated with neuropathy and peripheral arterial disease [6, 7]. Diabetic foot commonly develops as a result of long‐term disease progression and is influenced by factors such as aging, obesity, hypertension, smoking, and delayed care due to socioeconomic constraints. Additionally, financial hardship, cultural practices, limited education, and poor access to appropriate footwear and timely medical attention further elevate the risk of foot‐related complications [8].

Diabetic foot ulcers (DFUs) are a major contributor to morbidity with recurrence rates reaching up to 65% within 5 years [9]. Lower extremity amputations occur in a significant proportion of cases, and mortality rates remain high, underscoring the severity of the condition [10]. Lifetime risk of developing a DFU ranges from 19% to 34% and continues to rise, with increased disease duration and complexity [9].

Effective management of diabetic foot requires a comprehensive, patient‐centered approach, encompassing the assessment of vascular status, neuropathy, structural deformities, and pressure distribution [11].

Preventive strategies such as pressure offloading, appropriate footwear, and regular foot care are essential. Patient education plays a central role in improving self‐care practices and reducing complications associated with diabetic foot management. A multidisciplinary approach has been shown to significantly reduce amputation rates by 49%–85% and improve outcomes [12].

Physical therapists play an essential frontline role within the multidisciplinary team by playing a providing guidance on exercise prescription, functional assessment, gait training, pressure redistribution, and patient education. They are involved in early screening and prevention of complications, making them key stakeholders in diabetic foot care [13].

Prior studies have explored physiotherapists′ roles in promoting physical activity and managing chronic conditions. Despite this, there is a notable absence of literature addressing the knowledge, attitudes, and clinical practices of physiotherapists in India regarding diabetic foot care. Therefore, the present study was undertaken to identify gaps and inform future educational and clinical strategies [14].

## 2. Methods

### 2.1. Procedure

This national cross‐sectional online survey was conducted between January 2024 and March 2025 among physiotherapists practicing across India. The study was approved by the Institutional Scientific and Ethical Committee (Protocol No: IEC KMC MLR 12/2023/493), Kasturba Medical College, Manipal Academy of Higher Education, Mangalore.

Physiotherapists of any gender holding at least a bachelor′s degree, with a minimum of 2 years of experience in managing diabetic foot care and currently practicing in India, were included in the study. Exclusion criteria included physiotherapists not practicing in India, those with less than 2 years of clinical experience, and incomplete or duplicate responses.

A convenience sampling technique was employed due to the wide geographical distribution of participants and the online nature of the survey. The sample size was determined based on feasibility and anticipated response rates for online surveys among healthcare professionals. A total of 700 questionnaires were distributed, out of which 249 completed responses were received, yielding a response rate of 35.6%. To prevent duplicate responses, the survey was restricted to one response per email ID, and responses were screened for duplication prior to analysis.

#### 2.1.1. Questionnaire Development

The questionnaire used in this study was newly developed by the investigators based on existing literature and clinical guidelines related to diabetic foot care. It was not adapted from any previously validated instrument. The questionnaire was created using Google Forms and comprised 20 questions organized into four sections. These sections included respondents, demographic details (gender, educational qualifications, years of experience, and state of practice), and domains assessing knowledge, attitudes, and clinical practices related to diabetic foot care among physiotherapists practicing in India.

Section A consisted of five questions derived from established guidelines, intended to evaluate physiotherapists′ knowledge of diabetic foot care evaluation. Section B comprised five questions concerning physiotherapists′ attitude toward patient care, and Section C consisted of five questions designed to evaluate participants′ clinical practices in diabetic foot care. The questionnaire consisted of 20 items, including multiple‐choice and open‐ended questions, with the option for multiple responses. The estimated time required to complete the questionnaire was 10–15 min.

#### 2.1.2. Validity and Reliability

The survey questionnaire underwent a comprehensive review process involving feedback from a panel of five experts: Cardiopulmonary physiotherapists (*n* = 4) and a faculty member from the Department of Community Medicine (*n* = 1). All feedback was considered, and appropriate modifications were made.

A content validity index (CVI) was employed to assess the validity of the questionnaire and is widely regarded as one of the most commonly used methods for establishing the content validity of an instrument. The relevance of each item in the questionnaire was assessed using a 5‐point scale.

For a scale to demonstrate excellent content validity, items must meet Lynn′s (1986) criteria: an item‐level CVI (I‐CVI) of 1.00 for three to five experts and a minimum I‐CVI of 0.78 for 6–10 experts, along with a scale‐level CVI average (S‐CVI/Ave) of 0.90 or higher [15].

The final version of the survey was tailored to the recommendations and suggestions offered by the subject experts.

Content validation results indicated that all items achieved an I‐CVI of 1, demonstrating perfect agreement among the experts. The proportional relevance (PR) and universal agreement (UA) values were also found to be 1 for all items. Furthermore, the S‐CVI/Ave, calculated based on I‐CVI, PR, and UA, was 1, exceeding the recommended threshold and indicating excellent content validity of the instrument (Table 1).

**Table 1:** Content validity of questionnaire items using CVI based on expert ratings.

Sections	Question	Expert 1	Expert 2	Expert 3	Expert 4	Expert 5	Expert in agreement	I‐CVI	UA
A	Q1	5	4	5	5	5	5	1	1
	Q2	5	5	5	4	5	5	1	1
	Q3	5	5	5	5	5	5	1	1
	Q4	5	5	4	5	5	5	1	1
	Q5	4	4	5	5	5	5	1	1

B	Q6	5	5	5	5	5	5	1	1
	Q7	5	5	5	5	5	5	1	1
	Q8	5	5	5	5	5	5	1	1
	Q9	5	5	4	5	5	5	1	1
	Q10	4	5	5	5	5	5	1	1

C	Q11	5	5	5	5	5	5	1	1
	Q12	4	5	5	4	5	5	1	1
	Q13	4	5	4	4	5	5	1	1
	Q14	5	5	5	5	5	5	1	1
	Q15	5	5	5	5	5	5	1	1

	PR	1	1	1	1	1		S − CVI/Ave = 1	S − CVI/Ave = 1

Based on the obtained results, we can conclude that the S‐CVI/Ave calculated from I‐CVI, PR, and UA all met acceptable standards. Therefore, the questionnaire scale demonstrates adequate content validity.

#### 2.1.3. Data Collection Procedure

Email IDs of physiotherapists registered under the Indian Association of Physiotherapists (IAP) were shortlisted, and the questionnaire was distributed via email, as well as through WhatsApp and various online forums. A hyperlink directing participants to the survey web page was included in the distribution.

A succinct compilation of graduated physiotherapists, including their contact information, was assembled using data from the IAP website, physiotherapy departments, and pertinent Internet forums. These individuals were solicited to aid in disseminating the questionnaire, which was disseminated through various digital channels, including email, WhatsApp, and other online platforms. A hyperlink was provided, directing participants to the survey webpage, which included an informed consent form and a section for demographic details. The investigators provided prior information about the study′s scope and objectives. Participants proceeded to complete the questionnaire solely after granting their informed consent and meeting the requirements.

### 2.2. Data Analysis

As data collection was completed, the responses were transferred to Jamovi software (Version 2.6.23) for analysis. Descriptive statistics, including frequency, cumulative frequency, and percentage distributions, were calculated to summarize the categorical variables included in the study.

## 3. Result

### 3.1. Response

**Figure 1:**
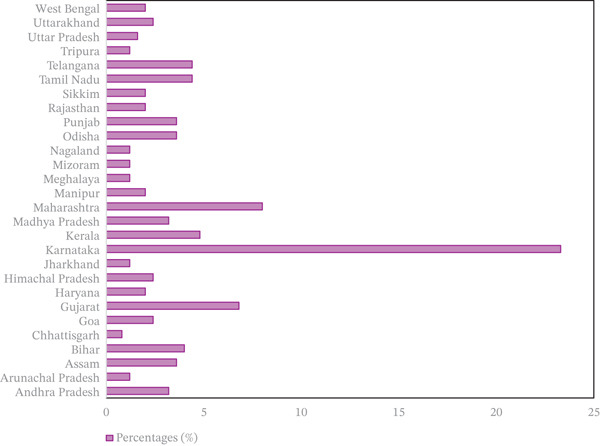
Response rate obtained from different states.

Out of 700 questionnaires distributed via email to physiotherapists throughout India, 249 participants responded and completed the survey. This made for a response rate of 35.57%. Responses were obtained from 28 states, including Andhra Pradesh, Arunachal Pradesh, Karnataka, Kerala, Assam, Bihar, Nagaland, Chhattisgarh, Goa, Gujarat, Haryana, Himachal Pradesh, Jharkhand, Madhya Pradesh, Maharashtra, Manipur, Meghalaya, Mizoram, Odisha, Punjab, Rajasthan, Sikkim, Tamil Nadu, Telangana, Tripura, Uttar Pradesh, Uttarakhand, and West Bengal. The majority of the responders were from Karnataka (*n* = 58 [23.3%]), Maharashtra (*n* = 20 [8.0%]), Gujarat (*n* = 17 [6.8%]), and Kerala (*n* = 12 [4.8%]) (Figure 1).

#### 3.1.1. Demographic Characteristics of the Participants

Table 2 presents the demographic information and educational qualifications of the participants. Of 249 respondents, the gender distribution was nearly balanced, wherein 122 were female (49.0%) and 127 were male (51.0%). The majority of academic qualifications were a bachelor′s degree (*n* = 128 [51.4%]), followed by a master′s degree (*n* = 106 [42.6%]) and a PhD (*n* = 15 [6.0%]). One hundred and eighty‐four had 2–5 years of experience, 6 had 5–10 years of experience, and 59 had > 10 years of experience with managing patients with diabetic foot conditions, of which 102 (41.0%) participants received specialized training or education related to treating diabetic foot issues/problems.

**Table 2:** Demographics and professional characteristics of the respondents.

Characteristics	*n*	%
Gender		
Female	122	49%
Male	127	51%
Educational qualifications		
Bachelor′s degree	128	51.4%
Master′s degree	106	42.6%
PhD	15	6.0%
Experience in the management of diabetic foot		
2–5 years	184	73.9%
5–10 years	6	2.4%
More than 10 years	59	23.7%
Received specialized training or education related to treating diabetic foot issues/problems		
Yes	102	41.0%
No	147	59.0%

#### 3.1.2. Knowledge

**Figure 2:**
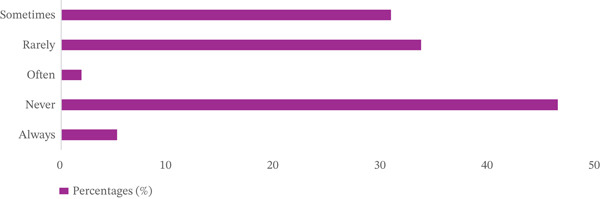
Response rate on willingness to treat a patient with an ABI value of 0.3.

Of the total 249 respondents, when asked about treatment decision‐making based on ABI values, 46.4% reported they would never treat a patient with an ABI of 0.3, 33.6% said they would rarely treat such cases, and 5.2% would always treat, indicating caution or limited understanding regarding ABI interpretation (Figure 2).

**Figure 3:**
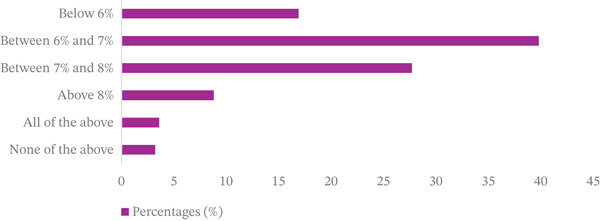
Responses received on recommended target HbA1c levels.

Regarding HbA1c targets for lowering the likelihood of diabetic foot–related issues, the most selected option was between 6% and 7% (39.8%), aligning with clinical guidelines. On the other hand, 27.7% chose between 7% and 8%, and 8.8% of participants chose above 8%, indicating some inconsistency in knowledge levels (Figure 3).

When asked about standard criteria followed to check foot sensations as an open‐ended question, the majority mentioned a various range of methods, with frequent emphasis on the Semmes–Weinstein monofilament test. Other commonly reported tools and techniques identified were “tuning fork test,” “pin‐prick test,” “vibration perception threshold” using a biothesiometer, and “ankle reflex testing.” Several participants also highlighted “proprioception,” “temperature sensation,” and “sensory modalities” such as touch, pain, and pressure. Certain responses noted the importance of comprehensive assessments, which included “dermatome testing” and “microfilament testing.”

**Figure 4:**
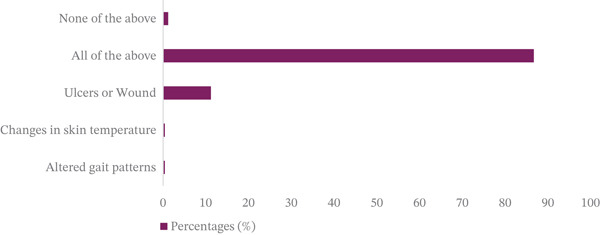
Responses on key signs indicating the need for specialized foot care in diabetes patients.

Participants were assessed to identify key signs indicative of the need for specialized foot care among diabetic individuals. The findings showed that most respondents (*n* = 216, 86.7%) selected the option “all of the above,” which included altered gait patterns, changes in skin temperature, and the presence of ulcers or wounds (Figure 4).

A range of comorbidities was reported by participants; which are summarized in Table 3. Since participants could report more than one comorbidity, the total frequency and percentage values may exceed the total number of participants.

**Table 3:** Common comorbidities reported in patients with diabetic foot.

**Comorbidities Reported**	**Frequency (n)**	**Percentages (%)**
Hypertension	68	27.3%
Peripheral neuropathy (diabetic neuropathy)	40	16.1%
Coronary artery disease/ myocardial infarction	50	20.1%
Obesity	38	15.3%
Dyslipidemia	11	4.4%
Chronic kidney disease	36	14.5%
Stroke	4	1.6%
Sleep apnea	6	2.4%
Foot ulcers/ diabetic foot	8	3.2%
Altered sensations/ numbness	11	4.4%

#### 3.1.3. Attitude

**Figure 5:**
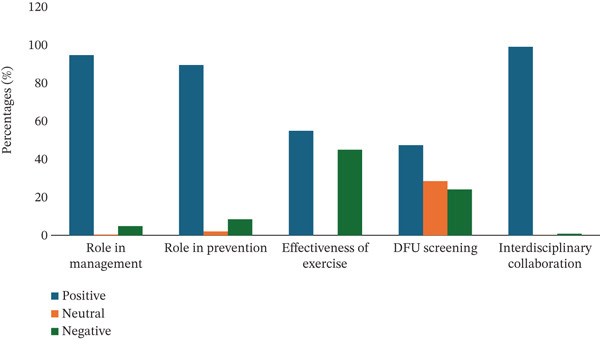
Frequency rate obtained on comparison of participants′ attitude toward diabetic foot care.

The survey investigated the role of physiotherapists in managing diabetes. The results indicated that a majority of respondents (94.8%, *n* = 236) believe that physiotherapists play a role in managing diabetes. A small portion of participants (4.8%, *n* = 12) were uncertain about the role of physiotherapists, while only 0.4% (*n* = 1) responded negatively (Figure 5).

The study also examined whether participants believed that physiotherapists played an important role in preventing diabetes. According to the responses, a large percentage of respondents (*n* = 233, 89.6%) believe that physiotherapists play an important role in diabetes prevention. Only a small proportion (*n* = 21, 8.4%) were unsure, and only 2% (*n* = 5) did not believe that physiotherapists played a role in prevention (Figure 5).

Participants were asked to assess the perceived effectiveness of exercise in the management of blood glucose levels among individuals with diabetes. Specifically, 27.3% (*n* = 68) rated exercise as “extremely effective,” 29.7% (*n* = 74) as “moderately effective,” 15.3% (*n* = 38) as “somewhat effective,” and 27.7% (*n* = 69) as “very effective” (Figure 5).

Participants were asked whether they routinely screen for DFUs during each clinical visit with diabetic patients. It was identified that 47.4% (*n* = 118) reported “yes” for performing routine screenings, 24.1% (*n* = 60) indicated that they are inconsistent (“not really”), and 28.5% (*n* = 71) reported “no” for screening for DFU during every visit (Figure 5).

Participants were asked to evaluate the significance of interdisciplinary collaboration for the effectiveness of diabetic foot health management. The responses were identified with 99.2% (*n* = 247) agreeing (“yes”) to the interdisciplinary collaboration, whereas only a small group (0.8%, *n* = 2) were uncertain (“not sure”), and none disagreed with the statement (Figure 5).

### 3.2. Clinical Practice

**Figure 6:**
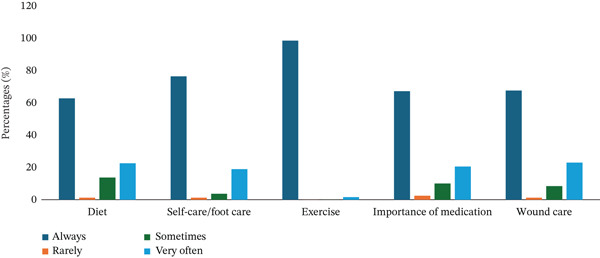
Frequency rate obtained on counseling diabetes management topics.

Respondents were asked about the frequency with which they provide dietary counseling as part of diabetes management. A substantial proportion reported consistent engagement in this aspect of care, with 62.7% (*n* = 156) indicating they “always” offer such counsel and an additional 22.5% (*n* = 56) doing so “very often.” A further 13.7% (*n* = 34) reported counseling patients “sometimes,” while only 1.2% (*n* = 3) did so “rarely.” Collectively, 98.9% of respondents engage in dietary counseling at least occasionally, with 85.2% doing so regularly (very often or always) (Figure 6).

Participants were also surveyed regarding how frequently they counsel patients on self‐care and foot care. A majority of respondents (76.3%, *n* = 193) reported “always” providing such counseling, while an additional 18.9% (*n* = 47) indicated they do so “very often.” Only a small fraction reported lower frequencies: 3.6% (*n* = 9) “sometimes” and 1.2% (*n* = 3) “rarely.” Overall, 98.8% of respondents guide self‐care and foot care at least occasionally, with 95.2% doing so regularly (Figure 6).

Participants were further asked about counseling regarding the role of exercise in diabetes management. Specifically, 98.4% (*n* = 245) stated they “always” counsel on this topic, while the remaining 1.6% (*n* = 4) do so “very often.” This unanimous engagement highlights the critical emphasis placed on promoting physical activity as a core intervention in diabetes care.

Similarly, when asked about counseling on the importance of medication adherence, 67.1% (*n* = 167) indicated they “always” offer guidance, with 20.5% (*n* = 51) doing so “very often.” An additional 10.0% (*n* = 25) reported counseling “sometimes,” while only 2.4% (*n* = 6) do so “rarely.”

The majority of respondents (90.4%) reported regularly counseling patients on wound care, with 67.5% doing so always and 22.9% very often. Only a small fraction (1.2%) provided such counseling rarely. Overall, 98.8% offer wound care advice at least sometimes.

**Figure 7:**
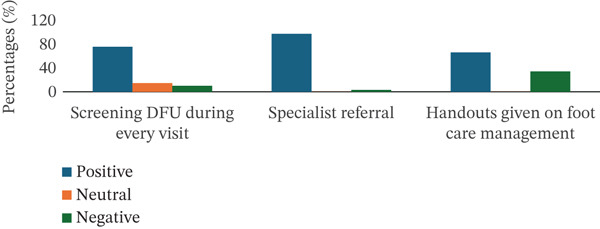
Frequency rate obtained on clinical practice regarding screening at every visit, referral, and handouts on foot care.

A majority of respondents (75.3%) reported that they screen for DFUs at every visit. However, 24.5% admitted to not doing so consistently or at all (Figure 7).

When asked if they would refer them to a podiatrist or general physician at times when abnormal signs were noticed, the majority (94%, *n* = 234) of respondents reported “yes” referring patients, whereas 6% (*n* = 15) reported “no” (Figure 7).

The majority (69.5%) of respondents reported using a comprehensive approach that includes clinical examination, as well as preventive and therapeutic care. Meanwhile, 21.7% relied on basic clinical examination alone, and 8.8% included callus removal and dressing. This variation indicates differing levels of depth in management strategies across practitioners (Figure 7).

**Figure 8:**
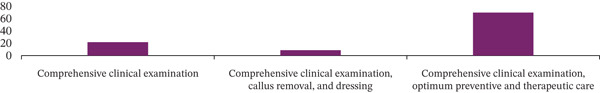
Response rate on approaches used in the management of diabetic foot.

Participants were asked if they would give the patients a handout on foot care management. It was identified that 65.9% (*n* = 164) reported giving out, whereas 34.1% (*n* = 85) of participants reported otherwise (Figure 8).

## 4. Discussion

This study intends to explore the awareness concerning diabetic foot care knowledge, the understandings, the attitudes, and current clinical practices among physiotherapists working in India.

India is known as the “diabetes capital of the world,” with millions of people affected by diabetes [3]. One of the most prevalent consequences of diabetes is DFU. Although several studies have evaluated patient awareness regarding diabetic foot issues [16, 17], there is a lack of literature specifically addressing the knowledge, attitudes, and practices (KAP) of Indian physiotherapists in diabetic foot care. Therefore, this study is aimed at assessing these domains and identifying existing gaps. While the study contributes important insights in the Indian context, the claim of being the first of its kind should be interpreted cautiously, as related work exists in other healthcare populations and international settings.

Our study revealed a strong level of agreement among respondents about the role of physiotherapists in both the management (94.8%) and prevention (89.6%) of diabetes. These findings are consistent with international trends where physiotherapists are increasingly integrated into multidisciplinary teams for chronic disease management. These findings resonate with Alhowimel et al. and Harris‐Hayes et al., who highlight physiotherapists as key stakeholders in diabetes care, particularly in exercise prescription, patient education, and preventive strategies [13, 18]. However, despite their strong affirmative attitude, gaps in knowledge and practice were evident, suggesting a disconnect between perceived role and actual clinical implementation.

Only 41% of participants reported having received formal training in diabetic foot care. A similar trend has been observed in studies among Canadian and Saudi Arabian physiotherapists [13, 19], indicating low levels of diabetes‐specific education. The discrepancy between perceived role and actual practice suggests systemic issues such as inadequate training and limited exposure during academic programs, which may hinder effective clinical application.

Knowledge assessment in our study showed mixed responses. While 86.7% correctly identified multiple clinical signs for foot complications, confusion persisted in interpreting clinical metrics like ABI and HbA1c levels. This particularly aligns with findings from other studies, where confidence in certain areas coexists with gaps in guideline‐based knowledge [20]. These inconsistencies highlight the need for strengthening structured education and improving familiarity with clinical guidelines to support better decision‐making. The observed variability in ABI interpretation may partly be explained by limited formal training, as indicated by the low proportion of participants with specialized education in diabetic foot care.

In terms of clinical practice, a large proportion (98.8%) reported engaging in patient counseling, but less than half conducted routine screening for foot ulcers, and 34.1% did not provide educational handouts, both of which are essential preventive measures. This suggests that while foundational knowledge may exist, its translation into regular, evidence‐based clinical practice remains limited. Similar inconsistencies were observed in the Saudi and Malaysian studies, underscoring a global need for reinforcing standard practices among physiotherapists [18, 20].

Importantly, experience appeared to play a role in knowledge retention and clinical confidence, as also reported by Zaworski et al., who found that years of clinical exposure significantly influenced physiotherapists′ understanding of diabetes‐related complications [21]. Likewise, our findings suggest that physiotherapists with greater clinical experience demonstrated better alignment between knowledge and practice. However, reliance solely on experience is insufficient, emphasizing the importance of structured training and continuous professional development.

The study also revealed a promising trend in interdisciplinary collaboration (99.2%), suggesting readiness to work alongside podiatrists, endocrinologists, and other healthcare professionals, reflecting growing awareness of the complex, multifactorial nature of diabetic foot care.

Despite these positive attitudes, several barriers remain. Limited access to specialized education, inconsistent clinical exposure, and lack of standardized national guidelines for physiotherapy in diabetic foot management were identified as contributing factors for suboptimal practice. These barriers are consistent with findings from other Indian studies where systemic limitations and gaps in education impact patient care outcomes [16]. Addressing these challenges through structured training programs, improved access to clinical guidelines, and enhanced integration into academic curricula is essential to improve consistency in practice.

### 4.1. Limitations

While this study offers valuable insights, certain limitations should be considered. The use of convenience sampling and online distribution via email and WhatsApp may limit the generalizability of the findings, as participation was restricted to physiotherapists with digital access. The data were self‐reported, which may introduce minor response bias.

## 5. Conclusion

This study highlights the disparity between physiotherapists′ awareness and clinical application of diabetic foot care in India. While most participants recognized their role in diabetic care, only a minority had received formal training. Key knowledge gaps and inconsistent screening practices were observed, indicating the need for improved clinical education and standardized guidelines. Experience appeared to positively influence practice quality.

Bridging the gap between awareness and application requires targeted educational initiatives and the establishment of standardized guidelines. Strengthening physiotherapists′ involvement through structured training, interprofessional collaboration, and curriculum integration can improve diabetic foot care outcomes and reduce complications like ulcerations, amputations, and associated healthcare burdens.

### 5.1. Clinical Significance

The findings from this study highlight a crucial gap in the awareness, attitudes, and practices related to the subject among the study population. By identifying specific areas of inadequate knowledge and prevalent misconceptions, the study offers valuable guidance for designing targeted educational initiatives. Physiotherapists typically acquire their knowledge through a combination of formal education, clinical experience, and continuing professional development activities, including educational programs, workshops, conferences, clinical training, research engagement, and reflective practices. The identified gaps can be addressed by strengthening professional education and ongoing training, promoting multidisciplinary and guideline‐based care, and enhancing early screening and patient education with a strong emphasis on evidence‐based practices. Addressing these issues requires an integrated approach that combines academic training with clinical practice–based learning, rather than relying on either approach alone.

### 5.2. Future Recommendations

Further research should adopt qualitative methods to better understand contextual factors, patient perceptions, and challenges affecting the adoption of best practices. It should also incorporate larger populations, digital education tools, policy changes, and the long‐term effectiveness of awareness and practice improvement programs.

## Funding

No funding was received for this manuscript.

## Conflicts of Interest

The authors declare no conflicts of interest.

## Data Availability

The data used in this study can be made available upon request to the corresponding author.

## References

[1] World Health Organization , Global Report on Diabetes, 2016, WHO.

[2] Harding J. L. , Pavkov M. E. , Magliano D. J. , Shaw J. E. , and Gregg E. W. , Global Trends in Diabetes Complications: A Review of Current Evidence, Diabetologia. (2019) 62, no. 1, 3–16, 10.1007/s00125-018-4711-2. 30171279

[3] International Diabetes Federation , IDF Diabetes Atlas, 2024, 10th edition, IDF.

[4] Saeedi P. , Petersohn I. , Salpea P. , Malanda B. , Karuranga S. , Unwin N. , Colagiuri S. , Guariguata L. , Motala A. A. , Ogurtsova K. , Shaw J. E. , Bright D. , Williams R. , and IDF Diabetes Atlas Committee , Global and Regional Diabetes Prevalence Estimates for 2019 and Projections for 2030 and 2045: Results from the International Diabetes Federation Diabetes Atlas, 9th Edition, Diabetes Research and Clinical Practice. (2019) 157, 107843, 10.1016/j.diabres.2019.107843, 31518657. 31518657

[5] American Diabetes Association , Classification and Diagnosis of Diabetes: Standards of Medical Care in Diabetes—2024, Diabetes Care. (2024) 47, no. Supplement 1, S15–S33. 10.2337/dc21-S002 33298413

[6] Armstrong D. G. , Boulton A. J. M. , and Bus S. A. , Diabetic Foot Ulcers and Their Recurrence, New England Journal of Medicine. (2017) 376, no. 24, 2367–2375, 10.1056/NEJMra1615439. 28614678

[7] Singh N. , Armstrong D. G. , and Lipsky B. A. , Preventing Foot Ulcers in Patients With Diabetes, JAMA. (2005) 293, no. 2, 217–228, 10.1001/jama.293.2.217. 15644549

[8] Yazdanpanah L. , Nasiri M. , and Adarvishi S. , Literature Review on the Management of Diabetic Foot Ulcer, World Journal of Diabetes. (2015) 6, no. 1, 37–53, 10.4239/wjd.v6.i1.37, 25685277. 25685277 PMC4317316

[9] Prompers L. , Huijberts M. , Apelqvist J. , Jude E. , Piaggesi A. , Bakker K. , Edmonds M. , Holstein P. , Jirkovska A. , Mauricio D. , Ragnarson Tennvall G. , Reike H. , Spraul M. , Uccioli L. , Urbancic V. , van Acker K. , van Baal J. , van Merode F. , and Schaper N. , High Prevalence of Ischaemia, Infection and Serious Comorbidity in Patients With Diabetic Foot Disease in Europe. Baseline results from the Eurodiale study, Diabetologia. (2007) 50, no. 1, 18–25, 10.1007/s00125-006-0491-1, 17093942. 17093942

[10] Lavery L. A. , Armstrong D. G. , Wunderlich R. P. , Mohler M. J. , Wendel C. S. , and Lipsky B. A. , Risk Factors for Foot Infections in Individuals With Diabetes, Diabetes Care. (2006) 29, no. 6, 1288–1293, 10.2337/dc05-2425. 16732010

[11] Apelqvist J. , Bakker K. , van Houtum W. H. , Nabuurs-Franssen M. H. , Schaper N. C. , and On Behalf of the International Working Group on the Diabetic Foot , International Consensus and Practical Guidelines on the Management and the Prevention of the Diabetic Foot, Diabetes/Metabolism Research and Reviews. (2000) 16, no. Supplement 1, S84–S92, 10.1002/1520-7560(200009/10)16:1+<::AID-DMRR113>3.0.CO;2-S, 11054895. 11054895

[12] Ragnarson Tennvall G. and Apelqvist J. , Prevention of Diabetes-Related Foot Ulcers and Amputations: A Cost-Utility Analysis Based on Markov Model Simulations, Diabetologia. (2001) 44, no. 11, 2077–2087, 10.1007/s001250100013, 11719840. 11719840

[13] Harris-Hayes M. , Combs S. A. , Pulley M. T. , Krauss M. J. , Bailey K. C. , Chiodo L. M. , and Mueller M. J. , Physical Therapists as Partners in Diabetes Prevention: An Observational Descriptive Study, Physical Therapy. (2020) 100, no. 3, 438–448.32043129

[14] American Diabetes Association , Disclosures: Standards of Care in Diabetes—2023, Diabetes Care. (2023) 46, no. Supplement 1, S281–S282, 10.2337/dc23-SDIS.

[15] Polit D. F. and Beck C. T. , The Content Validity Index: Are You Sure You Know What′s Being Reported? Critique and Recommendations, Research in Nursing & Health. (2006) 29, no. 5, 489–497, 10.1002/nur.20147, 16977646. 16977646

[16] Sudha B. G. , Umadevi V. , Shivaram J. M. , Chaluvanarayana H. C. , Sikkandar M. Y. , Belehalli P. , Ma S. , Hc C. , Sikkandar M. Y. , and Brioschi M. L. , Diabetic Foot Assessment and Care: Barriers and Facilitators in a Cross-Sectional Study in Bangalore, India, International Journal of Environmental Research and Public Health. (2023) 20, no. 11, 10.3390/ijerph20115929. PMC10252617 37297533

[17] Vibha S. P. , Kulkarni M. M. , Ballala A. B. K. , Kamath A. , and Maiya G. A. , Community-Based Study to Assess the Prevalence of Diabetic Foot Syndrome and Associated Risk Factors Among People With Diabetes Mellitus, BMC Endocrine Disorders. (2018) 18, no. 1, 10.1186/s12902-018-0270-2, 29940924. PMC6020220 29940924

[18] Alhowimel A. S. , Alshahrani A. A. , Abulaban A. A. , Althobeit A. M. , Alenazi A. M. , Alshehri M. M. , Alqahtani B. A. , and Alodaibi F. , Saudi Arabian Physical Therapists′ Knowledge, Attitudes, and Clinical Practice in Diabetes Prevention and Management, Diabetes, Metabolic Syndrome and Obesity. (2023) 16, 2967–2977, 10.2147/DMSO.S426949, 37767133. PMC10521925 37767133

[19] Doehring K. , Durno S. , Pakenham C. , Versi B. , and DePaul V. G. , Knowledge, Attitudes, and Current Practices of Canadian Physiotherapists in Preventing and Managing Diabetes, Physiotherapy Canada. (2016) 68, no. 3, 298–306, 10.3138/ptc.2015-63. 27909380 PMC5125468

[20] Krishnan P. , Krishnan K. , and Xiang C. J. , The Knowledge, Attitudes and Current Practices of Malaysian Physiotherapists in Preventing and Managing Type 2 Diabetes, Research Journal of Pharmacy and Technology. (2022) 15, no. 5, 2228–2235, 10.52711/0974-360X.2022.00370.

[21] Zaworski K. and Kubińska Z. , Level of Type 1 Diabetes Education of Physiotherapists Based on Selected Factors, Pediatric Endocrinology Diabetes and Metabolism. (2021) 27, no. 2, 109–116, 10.5114/pedm.2021.107164. 34514767 PMC10214974

